# Didymin protects pancreatic beta cells by enhancing mitochondrial function in high-fat diet-induced impaired glucose tolerance

**DOI:** 10.1186/s13098-023-01244-1

**Published:** 2024-01-03

**Authors:** Jingwen Yang, Ying Zou, Xiaoyu Lv, Jun Chen, Chen Cui, Jia Song, Mengmeng Yang, Huiqing Hu, Jing Gao, Longqing Xia, Liming Wang, Li Chen, Xinguo Hou

**Affiliations:** 1 Department of Endocrinology, Qilu Hospital of Shandong University, Cheeloo College of Medicine, Shandong University, 250012 Jinan, Shandong China; 2 Department of Endocrinology, The Second Hospital of Shandong University, Jinan, China; 3 Key Laboratory of Endocrine and Metabolic Diseases, Shandong Province Medicine & Health, Jinan, China; 4 Jinan Clinical Research Center for Endocrine and Metabolic Disease, Jinan, China; 5 Institute of Endocrine and Metabolic Diseases of Shandong University, Jinan, China; 6 National Key Laboratory for Innovation and Transformation of Luobing Theory, Jinan, China; 7 The Key Laboratory of Cardiovascular Remodeling and Function Research, Chinese Ministry of Education, Chinese National Health Commission and Chinese Academy of Medical Sciences, Jinan, China

**Keywords:** Didymin, High-fat diet, Impaired glucose tolerance, Pancreatic beta cell, Mitochondrial function

## Abstract

**Purpose:**

Prolonged exposure to plasma free fatty acids (FFAs) leads to impaired glucose tolerance (IGT) which can progress to type 2 diabetes (T2D) in the absence of timely and effective interventions. High-fat diet (HFD) leads to chronic inflammation and oxidative stress, impairing pancreatic beta cell (PBC) function. While Didymin, a flavonoid glycoside derived from *citrus* fruits, has beneficial effects on inflammation dysfunction, its specific role in HFD-induced IGT remains yet to be elucidated. Hence, this study aims to investigate the protective effects of Didymin on PBCs.

**Methods:**

HFD-induced IGT mice and INS-1 cells were used to explore the effect and mechanism of Didymin in alleviating IGT. Serum glucose and insulin levels were measured during the glucose tolerance and insulin tolerance tests to evaluate PBC function and insulin resistance. Next, RNA-seq analysis was performed to identify the pathways potentially influenced by Didymin in PBCs. Furthermore, we validated the effects of Didymin both in vitro and in vivo. Mitochondrial electron transport inhibitor (Rotenone) was used to further confirm that Didymin exerts its ameliorative effect by enhancing mitochondria function.

**Results:**

Didymin reduces postprandial glycemia and enhances 30-minute postprandial insulin levels in IGT mice. Moreover, Didymin was found to enhance mitochondria biogenesis and function, regulate insulin secretion, and alleviate inflammation and apoptosis. However, these effects were abrogated with the treatment of Rotenone, indicating that Didymin exerts its ameliorative effect by enhancing mitochondria function.

**Conclusions:**

Didymin exhibits therapeutic potential in the treatment of HFD-induced IGT. This beneficial effect is attributed to the amelioration of PBC dysfunction through improved mitochondrial function.

**Supplementary Information:**

The online version contains supplementary material available at 10.1186/s13098-023-01244-1.

## Introduction

Long-term consumption of a HFD leads to obesity, which is characterized by elevated levels of plasma free fatty acids (FFAs) levels, particularly saturated free fatty acids (SFAs). The elevated SFAs induce lipotoxicity in various organs [1], and palmitic acid (PA) is the most prevalent SFA in humans. PA has been associated with a higher hazard ratio for the development of diabetes [2] and is more toxic to rodents and human β-cells [3]. Prolonged exposure to FFAs leads to chronic inflammation and oxidative stress [4]. In PBCs, these factors contribute to enhanced basal insulin secretion and impaired glucose-stimulated insulin secretion (GSIS), which are the leading cause of IGT [5, 6]. Without timely and effective intervention, IGT will progress to type 2 diabetes [7].

Currently, bariatric surgery is considered the most effective method for the treatment of obesity [8]. However, it is associated with several negative outcomes such as perioperative chyloperitoneum and chylothorax, as well as postoperative complications including anemia, depression, fractures, malabsorption, etc. [9–11]. Only a few drugs such as acarbose, semaglutide, and liraglutide are used for the treatment of IGT, but they may cause side effects. Therefore, a safe and effective diet-based therapy is expected to be an option for IGT treatment.

Didymin (isosakuranetin 7-O-rutinoside) is an orally bioactive dietary flavonoid glycoside found in various citrus fruits such as oranges, lemons, mandarin, bergamot, grapefruit, chachi fruit, and citrus juices [12]. In recent years, studies have confirmed that Didymin showed promising biological activities including anticancer, antioxidant and neuroprotective, antinociceptive, hepatoprotective, anti-inflammatory, and cardiovascular activities [13–16]. The beneficial effects of many other kinds of flavonoids on beta cells of diabetic patients before dramatic dysfunction and degeneration have been studied extensively [17], but the protective effect of Didymin against lipotoxicity in PBCs remains unclear.

In this study, we aimed to investigate the protective effect of Didymin on beta cells in HFD-induced IGT through in vitro and in vivo experiments. Furthermore, we sought to elucidate the underlying mechanisms of its action.

## Materials and methods

### Cell culture and treatment

**Fig. 1:**
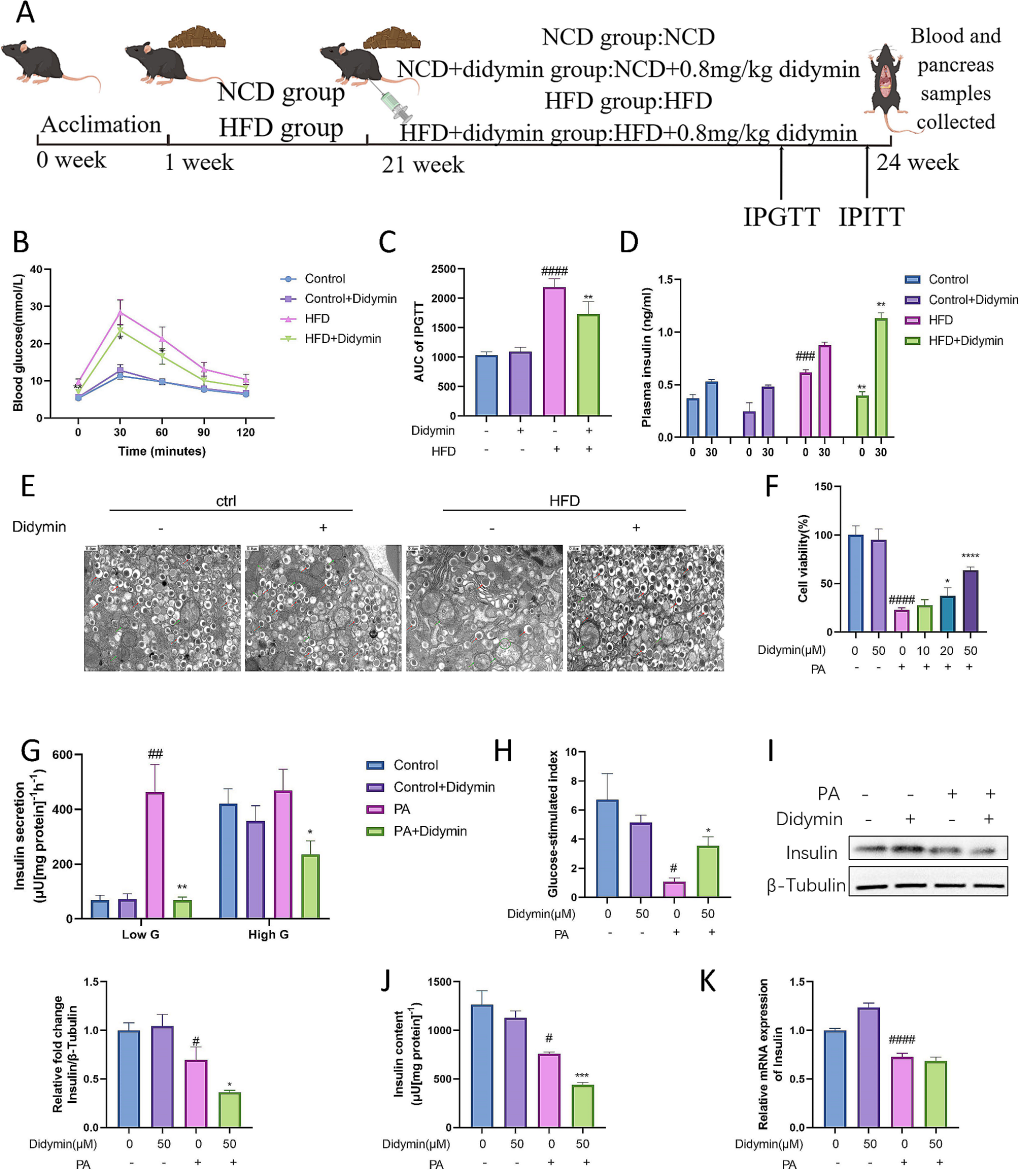
Didymin improves PBC insulin secretion function. (**A**) Graphical description of experimental design of this study. (**B**) Intraperitoneal glucose tolerance tests (IPGTT) (n = 5). (**C**) Area underneath IPGTT curve (AUC) (n = 5). (**D**) Plasma insulin responses (t = 0- and 30-min post) during IPGTT (n = 5). (**E**) Analysis of insulin granules in PBCs by electron microscopy, the red arrow indicates insulin granules, the green arrow indicates mitochondria, and the green circle points out damaged mitochondrial membrane. (**F**) Cell viability of INS-1 cells treated with PA and different concentrations of Didymin (n = 5). (**G**) Glucose-stimulated insulin secretion (GSIS) of INS-1 cells (n = 4). (**H**) Glucose-stimulated index (GSI) calculation. Intracellular insulin content of INS-1 cells after high glucose stimulation measured by (**I**) Western blot (n = 4) and (**G**) ELISA (n = 4). (**K**) Relative mRNA expression of insulin in INS-1 cells (n = 4). Data are expressed as mean ± SD. *P < 0.05, **P < 0.01, ***P < 0.001 DIO/PA vs. DIO + Didymin/PA + Didymin. # *P* < 0.05, ## *P* < 0.01, ### *P* < 0.001, #### *P* < 0.0001 control vs. DIO/PA.

INS-1 cells (Nanjing Medical University, PR China) were cultured in RPMI-1640 medium (Gibco, USA) containing 10% fetal bovine serum (FBS; Gibco, USA), 10mM Hepes (Sigma-Aldrich, St. Louis, MO), 1mM sodium pyruvate (Sigma-Aldrich, St. Louis, MO), 2mM L-glutamine (Gibco, USA) and 50 umol/L β-mercaptoethanol (Sigma-Aldrich, St. Louis, MO) at 37 °C with 5% CO2. Based on the concentration of Didymin used in the *in vitro* assay of previous studies and the effect of adding different concentrations of Didymin on the viability of INS-1 cells in our preliminary experiments (Fig. 1F), we chose a concentration of 50 µM for the *in vitro* assay. We also selected a PA concentration of 0.3 mM Based on the detection of cell viability (Fig.  S9). To investigate the effect of Didymin, INS-1 cells were treated as the following four groups: (1) Control groups: without Didymin or PA stimulated; (2) Control + Didymin groups: cells were incubated with Didymin (50 µM, MedChemExpress), without PA for 24 h; (3) PA groups: cells were treated with PA (0.3 mM) without Didymin for 24 h; (4) PA + Didymin groups: cells were treated with PA (0.3 mM) and Didymin (50 µM) simultaneously for 24 h. To clarify the role of mitochondrial function in Didymin-treated β cells, INS-1 cells were treated differently as follows: (1) PA groups: cells were treated with PA (0.3 mM) without Didymin or Rotenone for 24 h; (2) PA + Didymin groups: cells were treated with PA (0.3 mM) and Didymin (50 µM) simultaneously for 24 h; (3) PA + Rotenone groups: cells were treated with PA (0.3 mM) and Rotenone (0.1 µM) simultaneously for 24 h; (4) PA + Didymin + Rotenone groups: cells were treated with PA (0.3 mM) and Didymin (50 µM) and Rotenone (0.1 µM) simultaneously for 24 h.

### HFD-induced IGT mice model

Male C57BL/6J mice, aged four weeks, were obtained from Beijing SPF Biotechnology Co., Ltd., China. The mice were housed in a standard SPF facility at Shandong University, with four mice per cage at 22 °C and 12-h day and night cycle. After 1 week of acclimation, the mice were allocated at random into four groups (n = 8/group): (1) Control group: had a normal chow diet (12% fat, 20.6% protein, and 67.4% carbohydrate, SWS9102, Xietong Shengwu) for 20 weeks; (2) Control + Didymin group: had normal chow diet for 20 weeks and injected Didymin intraperitoneally with 0.8 mg/kg daily in the last three weeks; (3) DIO group: high-fat diet (60% fat, 20% carbohydrate, and 20% protein, XTHF60, Xietong Shengwu) for 20 weeks; (4) DIO + Didymin group: had HFD for 20 weeks and injected Didymin intraperitoneally with 0.8 mg/kg daily in the last three weeks. Based on previous research [16, 18]and the conversion of bioavailability between different administration methods [19], we chose a dosage of 0.8 mg/kg for intraperitoneal injection. One day following the final injection, mice were euthanized under anesthesia, blood and pancreas were collected. Each mouse pancreatic tissue was divided into two halves for histological and transmission electron microscopy (TEM) analysis. Figure 1a depicts the experimental design.

### Intraperitoneal glucose (IPGTT) and insulin tolerance test (IPITT)

For IPGTT, the mice were subjected to 16-h fasting followed by collecting blood from the tail vein before and at several time intervals (30, 60, 90, and 120 min) following intraperitoneal injection of 20% dextrose (2 g/kg body weight). Subsequently, Accu-Chek® Performa (Roche Life Science, USA) was employed to assess glucose levels. For insulin level measurements, blood samples were collected from the inner canthal vein of the eye during fasting and 30 min post-injection. For IPITT, the mice were subjected to 8-h fasting, followed by measuring tail vein blood glucose levels before and at several time intervals (30, 60, 90, and 120 min) following intraperitoneal insulin injection (0.75 U/kg body weight).

### Cell viability

The relative numbers of viable cells were determined using the Cell Counting Assay-8 kit (CCK-8; Beyotime Biotechnology, Shanghai, China) following the instructions.

### RNA-seq and real-time quantitative PCR (RT-qPCR) analysis

The isolation of total RNA was conducted employing Trizol reagent (Invitrogen, USA) following the protocols. The library construction and sequencing procedures were executed at Shenzhen BGI Genomics Co. The Dr. Tom multiple omics data mining system (https://biosys.bgi.com) was utilized for conducting data mining analysis. The Prime Script RT Reagent Kit (Cat. No. RR047A; Takara, Japan) was utilized to conduct reverse transcription. The SYBR Green PCR Kit (Cat. No. RR420A; Takara) was utilized to conduct RT-qPCR. The comparative CT (^2−ΔΔCt^) method was employed to determine gene expression changes, and quantification was accomplished by normalizing through Gapdh as a control. Table 1 lists the gene-specific primers utilized in PCR.

**Table 1:** qPCR primers

Name	5’ primer	3’ primer
28s	GATTCCCACTGTCCCTACC	AACCTCTCATGTCTCTTCACC
IL-1β	CAGCAATGGTCGGGACATAG	AGACTGCCCATTCTCGACAAG
IL-6	GCCTATTGAAAATCTGCTCTGG	ATTGCTCTGAATGACTCTGG
TNF-α	CAGCCGATTTGCCATTTCA	AGGGCTCTTGATGGCAGAGA
Insulin	ATCTTTGGTCTGGCTCCCATG	TTTCCCGTTCACCGTCCAC

### Glucose-stimulated insulin secretion (GSIS)

Following a 30 min preincubation in glucose-free KRB HEPES buffer, INS-1 cells went through 1 h incubation in KRB HEPES buffer that contained low glucose (LG) concentration (2.5 mmol/L), followed by another 1 h incubation in high glucose (HG) concentration (25 mmol/L). The rat insulin ELISA kit (Lianke Biotechnology, Shanghai, China) was utilized to quantify insulin levels. Following the experiment, the total protein concentration was evaluated through the bicinchoninic acid (BCA) method and the BCA protein assay reagent (Beyotime Biotechnology, Shanghai, China) to assess the concentration of insulin.

### Enzyme-linked immunosorbent assay (ELISA)

Mouse serum insulin (80-INSMSUE01, ALPCO), INS-1 cell insulin, and pro-inflammatory cytokines (Lianke Biotechnology, Shanghai, China) were tested using ELISA kits per instructions.

### Western blot analysis

The INS-1 cells were subjected to lysis using radioimmunoprecipitation assay (RIPA) lysis buffer (P0013B, Beyotime, Shanghai, China), and the protein concentration was detected using the bicinchoninic acid (BCA) method (Beyotime, China). Use SDS-PAGE to separate the proteins. Following this, the proteins were transferred onto polyvinylidene difluoride (PVDF) membranes (IPVH00010 0.45 μm, Millipore, USA). The membranes were then subjected to a 1 h blocking procedure using 5% skim milk at room temperature (RT) and overnight incubation in specific primary antibodies at 4 °C. Following 1 h incubation with horseradish peroxidase-conjugated secondary antibodies at RT, enhanced chemiluminescence was employed for visualizing the proteins. Table 2 lists the primary antibodies used.

**Table 2:** Antibodies

Antibody	Source	Vendor	Catalog No.
Insulin	Rabbit	ABcam	ab181547
TNF-α	Rabbit	Bioss	bs-2081R
IL-6	Rabbit	Bioss	bs-4539R
IL-1β	Rabbit	Bioss	bs-0812R
PARP	Rabbit	Cell Signaling Technology	#9532
Cleaved PARP (Asp214)	Rabbit	Cell Signaling Technology	#94,885
Bax	Rabbit	Cell Signaling Technology	#2772
Bcl-2	Mouse	ImmunoWay	YM3041
caspase-3	Rabbit	Cell Signaling Technology	#9662
cleaved caspase-3 (Asp175)	Rabbit	Cell Signaling Technology	#9661
NRF1	Rabbit	Proteintech	12482-1-AP
TFAM	Rabbit	Proteintech	22586-1-AP
NDUFB8	Rabbit	Proteintech	14794-1-AP
MT-CO2	Rabbit	Cell Signaling Technology	#31,219
SDHB	Rabbit	Cell Signaling Technology	#92,649
HSP90	Rabbit	Proteintech	13171-1-AP
β-Tubulin	Rabbit	Proteintech	10068-1-AP

### MitoTracker Green staining

Mitochondria went through staining using MitoTracker Green probes (Beyotime Biotechnology) per instructions. The study utilized a high-speed confocal platform (Dragonfly 200, Andor, UK) for capturing fluorescent images. The fluorescence intensities were corrected for the protein concentration.

### Seahorse analysis

The oxygen consumption rate (OCR) was measured employing an Agilent Seahorse Bioscience XF24-3 Extracellular Flux Analyzer (Agilent, Santa Clara, CA). The experimental procedures involved the utilization of 1.5 µM oligomycin, 2 µM carbonyl cyanide-4-(trifluoromethoxy) phenylhydrazone (FCCP), and 0.5 µM antimycin A/Rotenone per instructions. Data normalization was performed based on the total cell count. Results were subjected to analysis in WAVE software (2.6.3) and processed through the XF Mito Stress Test Report.

### Flow Cytometry

Flow cytometry was utilized for detecting cell apoptosis employing an annexin V-FITC apoptosis detection kit (BestBio, Shanghai, China) per instructions. After Annexin V-FITC and PI staining, early and late cell apoptotic activities were detected using a Beckman Coulter Gallios flow cytometer.

### TUNEL

The cells underwent fixation using a 4% paraformaldehyde and permeabilization using 0.3% Triton X-100. Subsequently, the cells were subjected to staining using a TUNEL reaction solution (Servicebio, G1502-100T). Afterward, the fluorescence microscope was utilized to observe the images, and the proportion of total cells that exhibited positive staining was calculated using Image J software (NIH, Bethesda, United States).

### Histological and TEM analysis

For histological investigation, the pancreas was washed with PBS, followed by 24 h fixation in 4% paraformaldehyde, and then dehydrated. Subsequently, the specimens were embedded in paraffin and sectioned into 4 μm tissue slices. To conduct immunofluorescence staining, the slides were subjected to dewaxing followed by performing antigen retrieval through antigen unmasking buffer. Following a 30-min blocking at RT in a protein-blocking solution (10% normal goat serum), the slides went through overnight incubation with primary antibody at 4 °C. Subsequently, the slides were subjected to 60 min incubation with a fluorescent secondary antibody at RT, followed by 5 min staining with DAPI. Fluorescence imaging was conducted and recorded utilizing a fluorescence microscope (Olympus BX53, Japan).

The beta cell ultrastructure was examined utilizing TEM (JEM-1200EX). The pancreases were treated with a 2.5% glutaraldehyde solution for fixation. Following that, the specimens received two rounds of washing using 0.1 M phosphate buffer for 30 min each. Subsequently, they were fixed for 2 h using 1% OSO4 and subjected to dehydration through a series of ethanol concentrations, 50%, 70%, 80%, and 90%. The specimens were subjected to embedding with an epoxy resin mixture, followed by cutting the resultant blocks using an ultramicrotome.

### Statistical analysis

All data were analyzed with GraphPad Prism 8.0 (GraphPad Software, San Diego, Canada) and expressed as means ± SD. The statistical analyses employed in this study included the utilization of the Student’s t-test to examine differences between two groups, and the one-way ANOVA to assess differences among more than two groups. Adjusted *p* < 0.05 indicated a significant difference.

## Results

### Didymin improves PBC insulin secretion function

To assess the potential of Didymin in enhancing PBC function, we utilized the HFD-induced IGT mice and PA-induced INS-1 cells. IPGTT results showed that the HFD mice exhibited a significant IGT compared to the control group. However, Didymin administration significantly reduced the blood glucose level at 30 and 60 min after glucose injection in IGT mice, indicating the effective mitigation of the disruption of glucose metabolism (Fig. 1B-C). The improved glucose tolerance suggests that Didymin may improve insulin secretion in IGT mice or improve insulin sensitivity in peripheral tissues [20, 21]. To further explore the physiological processes improved by Didymin, we examined plasma insulin levels after glucose injection and performed an IPITT test. The decrease in fasting plasma insulin levels may be attributed to the decrease in fasting plasma glucose levels and may also suggest an increase in insulin sensitivity in IGT mice (Fig. 1D). Moreover, the increases in plasma insulin level 30 min after glucose injection indicates an improvement in early insulin response, reflecting the improved ability of the pancreas to release insulin under glucose stimulation (Fig. 1D). The results of IPITT further support the improvement in insulin sensitivity in IGT mice with Didymin treatment (Fig. S1). Transmission electron microscopy images of mouse PBCs (Fig. 1E) and the results of immunofluorescent staining of insulin in pancreatic sections Fig. S2) revealed that Didymin intervention alleviated the decrease in insulin secretory granules and insulin content in fasting IGT mice. This finding is consistent with the results of plasma insulin level, where more insulin secretion occurs in the fasting state of IGT mice due to insulin resistance, resulting in a decrease in intracellular insulin granules. Conversely, impaired glucose-stimulated insulin secretion was observed due to insufficient intracellular insulin granules. Therefore, Didymin improves the insulin secretion function of PBCs and enhances glucose metabolism in IGT mice.

Furthermore, we investigated the effect of Didymin on INS-1 cells. 0.3 mM PA was used to simulate lipotoxicity in INS-1 cells. Didymin treatment resulted in a concentration-dependently increase in cell viability compared to PA-treated INS-1 cells, as determined by the CCK8 assay (Fig. 1F). Based on its significant therapeutic effect, we selected a concentration of 50µM Didymin for the subsequent experiments. We examined the insulin secretion function of INS-1 cells using glucose-stimulated insulin secretion (GSIS) assays. Insulin release was increased under low glucose stimulation and decreased under high glucose stimulation in the PA group compared to the control group. However, co-treatment with PA and Didymin reversed these effects, with reduced insulin secretion under low glucose stimulation and increased insulin secretion under high glucose stimulation compared to the PA treatment group (Fig. 1G). The glucose-stimulated index (GSI), which reflects the insulin secretion ratio of PBCs stimulated by high glucose, indicating the insulin release ability of INS-1 cells under glucose stimulation, was decreased in the PA treatment group but restored by co-treatment with PA and Didymin (Fig. 1H). Furthermore, we measured the intracellular insulin content after high glucose stimulation in INS-1 cells using Western blot and ELISA assays. The results showed that PA stimulation decreased insulin content, while co-stimulation with PA and Didymin further reduced insulin content (Fig. 1I-J). Furthermore, RT-qPCR results showed that PA and Didymin stimulation did not significantly affect gene expression of Insulin (Fig. 1K). These results indicate that Didymin does not affect the insulin synthesis level of PBCs. Under low glucose conditions, Didymin reduces the excessive insulin secretion induced by PA in PBCs, leading to an increase in intracellular insulin storage. As a result, when exposed to high glucose stimulation, PBCs can release a large amount of insulin in the first phase, thereby significantly improving the function of PBCs.

### Didymin protects INS-1 cells against PA-induced lipotoxicity by enhancing mitochondrial function, attenuating inflammation, and inhibiting apoptosis

**Fig. 2:**
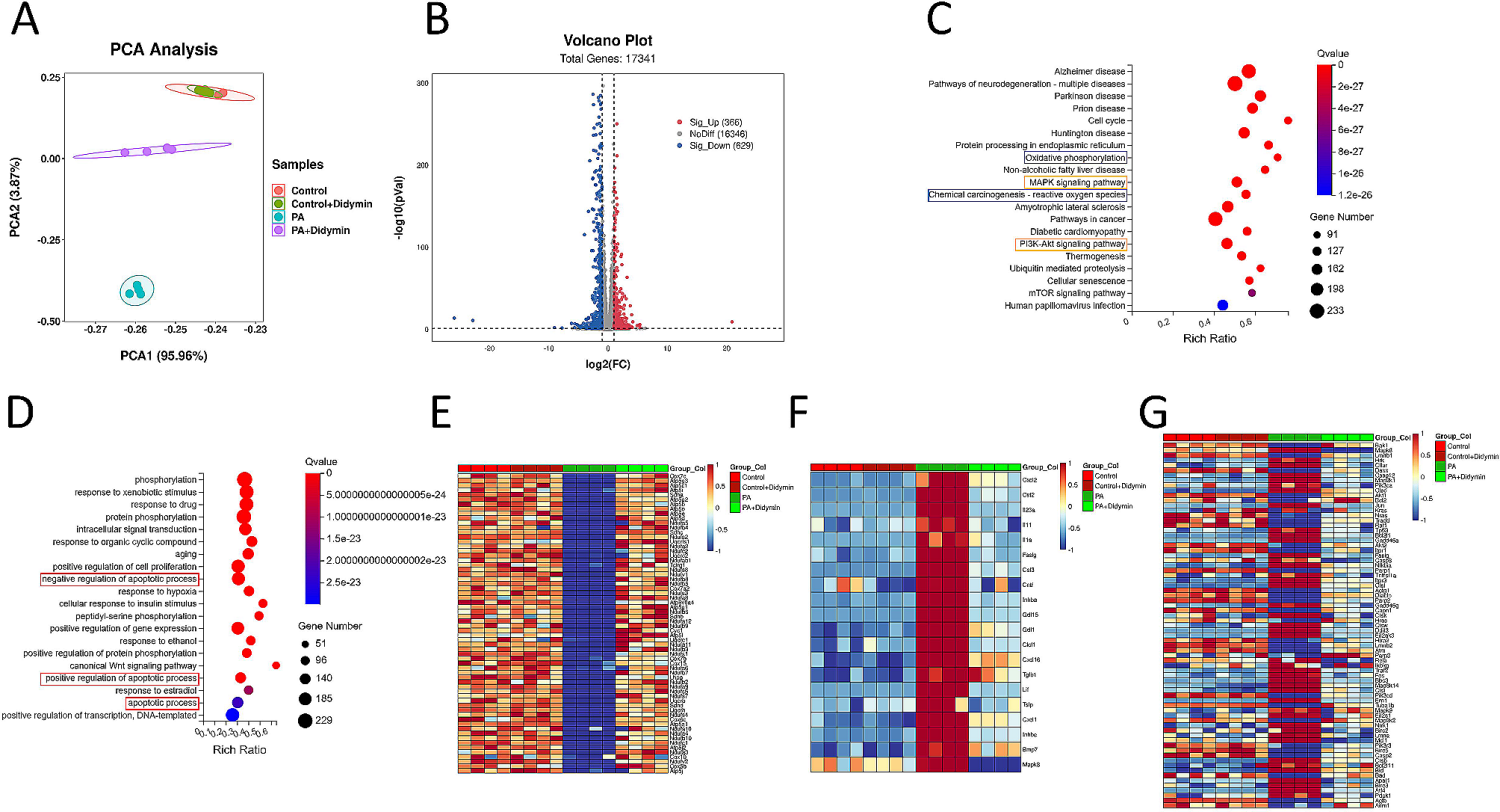
Transcriptomic analysis reveals the key differential targets in Didymin-treated INS-1 cells. (**A**) Principal component analysis (PCA) of the RNA-sequencing data of INS-1 cells. (**B**) Volcano-plot of RNA-seq results for PA + Didymin vs.PA. (**C**) KEGG analysis of the enrichment pathways. (**D**) GO Process (GO-P) analysis. Heatmaps of gene expression profiles related to (**E**) mitochondrial function, (**F**) inflammation, and (**G**) apoptosis based on the RNA-seq data set. n = 4 per group

To elucidate the mechanisms underlying the effects of Didymin on PBCs, we conducted RNA sequencing in INS-1 cells. Unsupervised principal component analysis (PCA) and hierarchical clustering analysis clearly distinguished the PA-treated INS-1 cell samples from the samples co-treated with PA and Didymin (Fig. 2A). We identified 995 differentially expressed genes (DEGs), with 366 genes upregulated and 629 genes downregulated in the group co-treated with PA and Didymin compared to the group treated with PA alone (Fig. 2B). Kyoto Encyclopedia of Genes and Genomes (KEGG) pathway analysis revealed that genes related to mitochondria function and inflammation were enriched (Fig. 2C). Gene Ontology Process (GO-P) analysis demonstrated the enrichment of genes related to the apoptotic process (Fig. 2D). Since both positive and negative regulators of apoptosis were enriched at the same time, we further analyzed their expression. Compared with the PA group, the expression of positive regulators in the PA + Didymin group was mostly decreased (Fig. S3A), and the expression of negative regulators was mostly increased (Fig. S3B), suggesting that Didymin inhibited apoptosis. Moreover, heatmaps of DEGs illustrated significant alternations in the expression of genes involved in mitochondrial function, inflammation, and apoptosis pathways following PA treatment, with a reduction in such changes upon Didymin intervention (Fig. 2E-G). Collectively, these results indicate that Didymin treatment protects PBCs against lipotoxicity by improving mitochondrial function, suppressing inflammation, and inhibiting apoptosis.

### Didymin protects PBCs against lipotoxicity by enhancing mitochondrial function

**Fig. 3:**
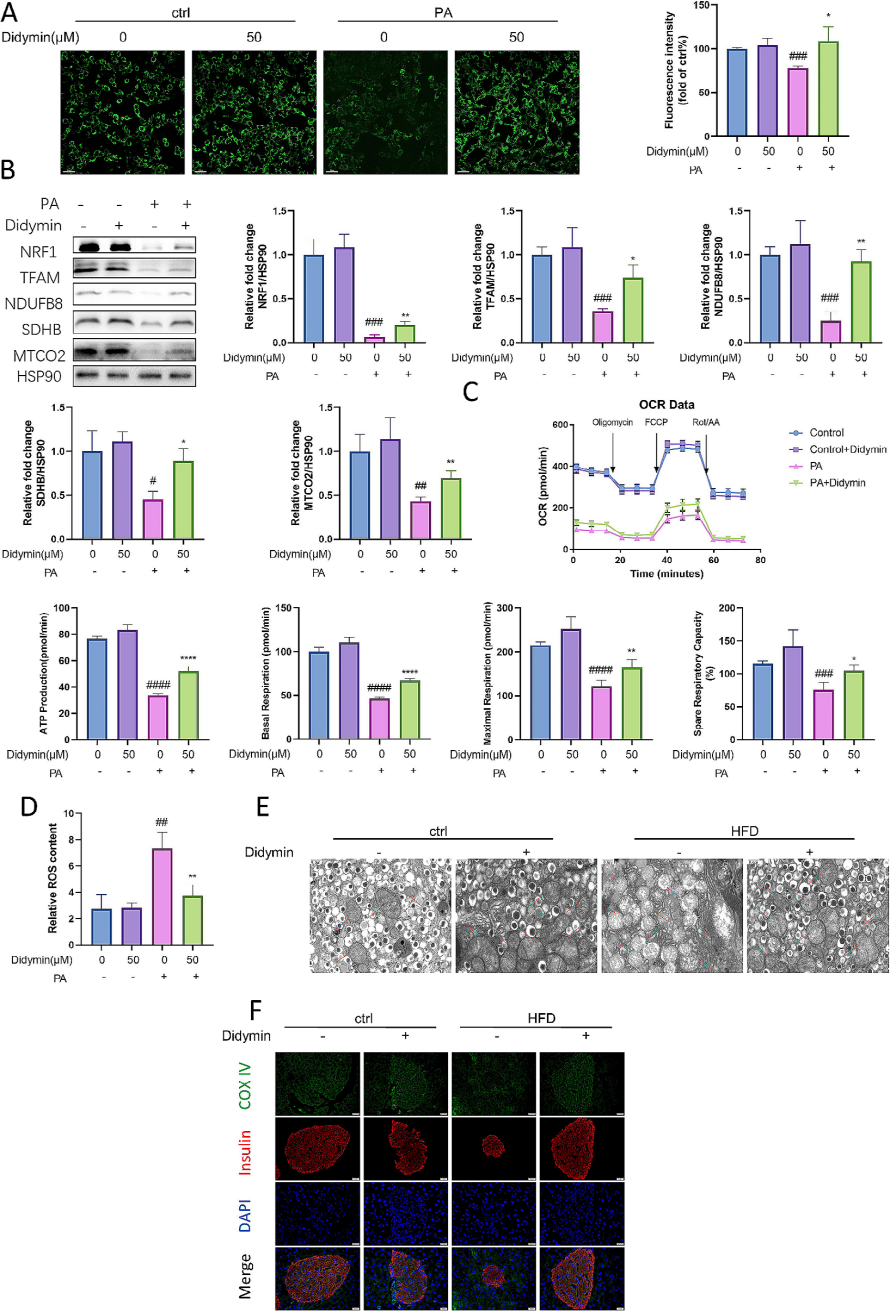
Didymin improves mitochondrial function in PBCs. (**A**) MitoTracker Green staining for mitochondrial content in INS-1 cells. Scale bar = 30 μm. (n = 4) (**B**) Western blot analysis of NRF1, TFAM, NDUFB8, SDHB and MTCO2 in INS-1 cells (n = 3). (**C**) Mitochondrial oxygen consumption ratio (OCR) of INS-1 cells (n = 4). (**D**) ROS concentration in INS-1 cells (n = 4). (**E**) Analysis of mitochondrial structure of PBCs in mice by electron microscopy. Scale bar = 0.6 μm. (**F**) Immunofluorescence staining of COX-IV (green) and insulin (red) in pancreas. Scale bar = 20 μm. Data are expressed as mean ± SD. *P < 0.05, **P < 0.01, ****P < 0.0001 PA vs. PA + Didymin. # *P* < 0.05, ## *P* < 0.01, ### *P* < 0.001, #### *P* < 0.0001 control vs. PA.

PA-induced lipotoxicity is accompanied by mitochondrial loss and dysfunction. To investigate the effects of Didymin on mitochondria, we performed MitoTracker Green staining and found that PA-induced lipotoxicity leads to mitochondrial depletion, while Didymin treatment elevated mitochondrial content in the PA-treated group (Fig. 3A). Notably, Didymin upregulated the expression of transcription factors involved in mitochondrial biogenesis, NRF1 and TFAM, as well as components of the electron transport chain (ETC), NDUFB8, SDHB, and MTCO2, in PA-treated INS-1 cells (Fig. 3B). Moreover, Didymin remarkably reversed PA-induced suppression of OCR, as indicated by higher levels of ATP production, basal respiration, maximum respiration, and spare respiratory capacity (Fig. 3C). Additionally, Didymin suppressed PA-induced ROS generation (Fig. 3D). Since the synthesis pathway of ROS includes both mitochondrial electron transport chain and NOX [22], we detected the cellular NOX activity, the results showed that Didymin did not significantly affect the activity of NOX (Fig. S4). These results indicate that Didymin improves the mitochondrial function of INS-1 cells.

To further validate the mechanisms of Didymin in the treatment of IGT mice in vivo, we examined the ultrastructure and ETC components of mitochondria in PBCs derived from wild-type (WT) and Didymin-treated mice. Electron microscopy results showed a normal alignment and intact mitochondrial cristae structure in PBCs from WT mice, but shrunken mitochondria, decreased cristae, and ruptured outer membrane in PBCs from IGT mice. However, Didymin treatment ameliorated changes in mitochondrial structure and also increased the number of mitochondria. The mitochondria in the PBCs of HFD mice are swollen and larger, the density of mitochondria is significantly reduced, abnormal granular or massive structure is observed, the damage of mitochondrial membrane is also shown in the green circle (Fig. 1E), suggesting mitochondrial damage. The mitochondria in PBCs of HFD + didymin mice were slightly swollen, and the density was not significantly reduced, but the mitochondrial crista structure was still visible (Figs. 1E and 3E). Immunofluorescence assay further demonstrated that lipotoxicity inhibited the expression of COX-IV, a component of ETC, in PBCs of IGT mice, while Didymin treatment potentiated COX-IV expression (Fig. 3F).

Overall, these findings indicate that Didymin enhanced mitochondrial biogenesis and function in PBCs, protecting them against lipotoxicity induced by PA.

### Didymin attenuates inflammation to protect PBCs against lipotoxicity

**Fig. 4:**
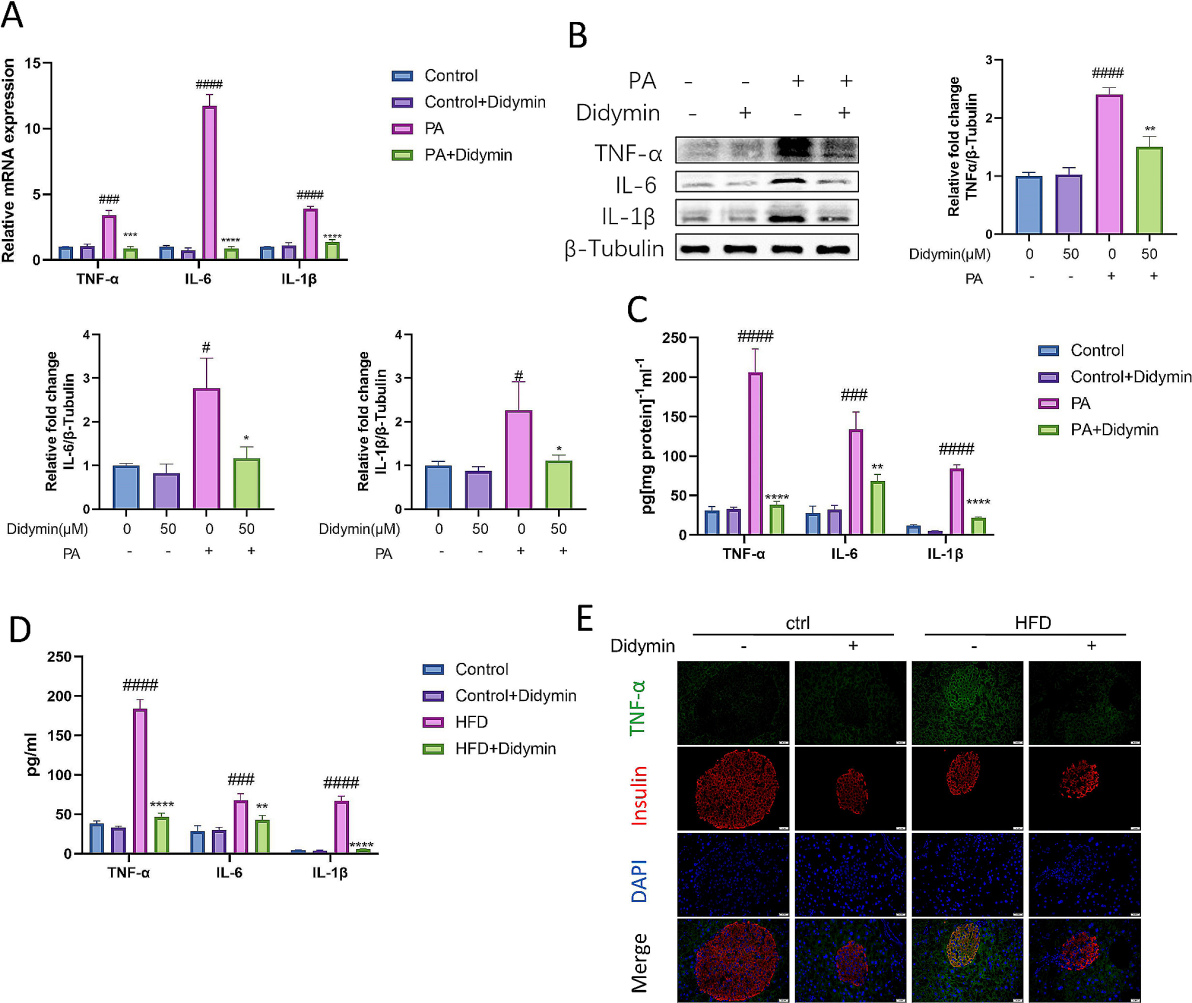
Didymin inhibits inflammation in PBCs. (**A**) Relative mRNA expression of TNF-α, IL-6 and IL-1β. The concentrations of TNF-α, IL-6 and IL-1β in INS-1 cells measured by (**B**) Western blot and (**C**) ELISA. (**D**) Immunofluorescence staining of TNF-α (green) and insulin (red) in pancreas. Scale bar = 20 μm. (**E**) Serum TNF-α, IL-6 and IL-1β measured by ELISA. Data are expressed as mean ± SD (n = 4). *P < 0.05, **P < 0.01, ***P < 0.001, ****P < 0.0001 PA/DIO vs. PA + Didymin/DIO + Didymin. # *P* < 0.05, ### *P* < 0.001, #### *P* < 0.0001 control vs. PA/DIO.

To investigate the effect of Didymin on inflammation in INS-1 cells stimulated by PA, we performed real-time qPCR, Western blot, and ELISA assays to assess the levels of pro-inflammatory cytokines. We found that Didymin treatment could effectively abolish PA-induced upregulation of TNF-α, IL-1β, and IL-6 at both the transcriptional and protein levels in INS-1 cells (Fig. 4A-C), indicating that Didymin exerts an anti-inflammatory property. We further examined whether Didymin regulates activation of the NF-kB pathway. Western blot results showed that the phosphorylation level of p65 and expression level of TLR9 in INS-1 cells was decreased after the addition of didymin, while the level of TLR4 was not significantly changed (Fig. S5). These suggest that Didymin may inhibit the activation of the NF-kB pathway by affecting intracellular stimulus, such as mitochondrial DNA leakage and ROS production [23].

Furthermore, we observed a significant increase in TNF-α level in PBCs of IGT mice, which was significantly decreased upon Didymin treatment (Fig. 4D). Additionaly, Didymin considerably decreased the concentrations of TNF-α, IL-6, and IL-1β in plasma compared to the IGT group (Fig. 4E). These results indicated that Didymin inhibits inflammation in PBCs to protect them against lipotoxicity.

### Didymin protects PBCs against lipotoxicity by inhibiting apoptosis

**Fig. 5:**
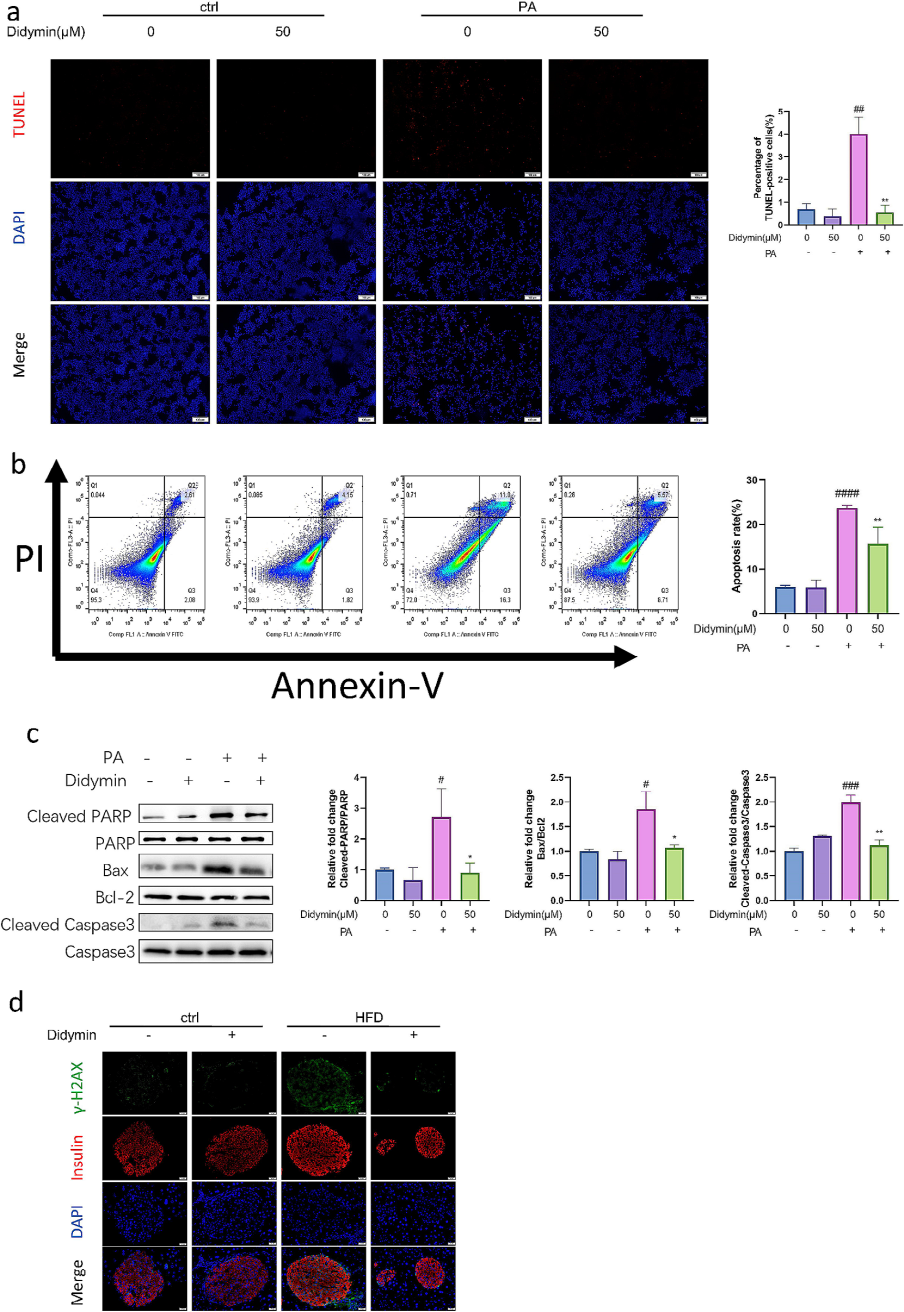
Didymin inhibits apoptosis in PBCs. (**A**) Representative images of TUNEL staining in INS-1 cells. Scale bar = 100 μm. And quantification of the percentage of TUNEL positive cells (n = 3) (**B**) Apoptosis analysis of INS-1 cells by flow cytometry (n = 4). (**C**) Western blot analysis of the cleaved-PARP, PARP, Bax, Bcl2, cleaved-caspase3, and caspase3 proteins in INS-1 cells (n = 3). (**D**) Immunofluorescence staining of γ-H2AX (green) and insulin (red) in pancreas. Scale bar = 20 μm. Data are expressed as mean ± SD. *P < 0.05, **P < 0.01, ****P < 0.0001 PA vs. PA + Didymin. # *P* < 0.05, ### *P* < 0.001, #### *P* < 0.0001 control vs. PA.

GO analysis of our RNA-seq data demonstrated that Didymin reduced the expression of apoptotic related genes (Fig. 2D). To further confirm the function of Didymin in this process, we employed TUNEL staining and flow cytometry analysis of the annexin-V, as well as PI staining. The results showed that number of apoptotic cells increased notably following PA treatment, but was down-regulated by Didymin (Fig. 5A, B). Furthermore, key molecules involved in the apoptosis pathway, including Bax, Bcl-2, PARP, and Caspase3, were analyzed to assess the impact of Didymin in this process [24]. Western blot analysis demonstrated that Didymin reduced the ratios of cleaved-PARP/PARP, Bax/Bcl2, and Cleaved Caspase3/Caspase3 (Fig. 5C).

We next stained pancreatic beta cells with γ-H2AX (phosphorylated H2AX), a DNA double-strand break marker, to detect unrepaired DNA damage, which indicates apoptotic DNA fragmentation. Immunofluorescent staining results showed a higher level of positive staining apoptotic PBCs in IGT mice, which was significantly reduced by Didymin (Fig. 5D). We also measured the islet area in the pancreatic tissue sections of each group of mice. The results showed that the islet area of the HFD group was slightly smaller than that of the Control group, while the islet area of the HFD + Didymin group was larger than that of the HFD group (Fig. S6). This suggests that Didymin improves the apoptosis of PBCs in HFD mice.

Taken together, these results proved evidences that Didymin exerts significant therapeutic effects against lipotoxicity-induced apoptosis in PBCs.

### Didymin improves insulin secretion function, attenuates inflammation, and inhibits apoptosis by enhancing mitochondrial function in INS-1 cells

**Fig. 6:**
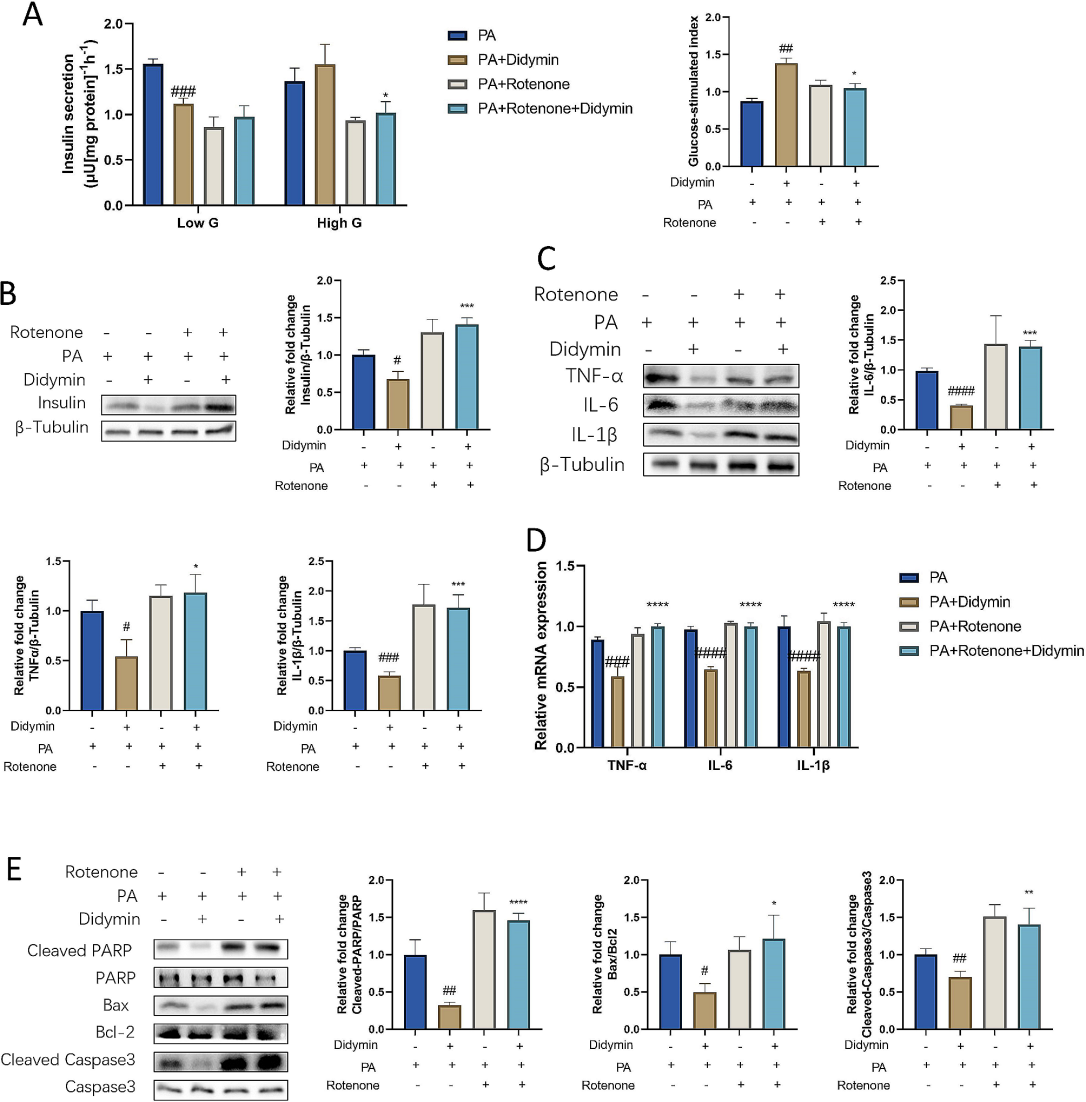
Didymin attenuates inflammation, improves GSIS and inhibits apoptosis through enhancing mitochondrial function. (**A**) Glucose-stimulated insulin secretion (GSIS) and Glucose-stimulated index (GSI) calculation. (**B**) Western blot measurement of intracellular insulin content. (**C**) Western blot measurement of TNF-α, IL-6 and IL-1β in INS-1 cells. (**D**) Relative mRNA expression of TNF-α, IL-6 and IL-1β. (**E**) Western blot analysis of the cleaved-PARP, PARP, Bax, Bcl2, cleaved-caspase3, and caspase3 proteins in INS-1 cells. Data are expressed as mean ± SD (n = 3). *P < 0.05, **P < 0.01, ***P < 0.001, ****P < 0.0001 PA + Didymin vs. PA + Didymin + Rotenone. # *P* < 0.05, ## *P* < 0.01, ### *P* < 0.001, #### *P* < 0.0001 PA vs. PA + Didymin.

Mitochondria play a crucial role in sensing lipotoxic stimuli and regulating cellular physiological processes [25–27]. To verify whether the protective effect of Didymin is mediated by enhancing mitochondrial function, INS-1 cells were treated with Rotenone, a mitochondrial electron transport chain complex I inhibitor, together with Didymin. Then, we evaluated its GSIS function, inflammation cytokine levels, and apoptosis levels. While Didymin improved insulin secretion in response to high glucose (HG) stimulation, Rotenone counteracted this effect (Fig. 6A). Western blot results further indicated that more insulin remained in the cells, and can’t be secreted following high glucose stimulation when the cells were treated with Rotenone (Fig. 6B). Inhibition of mitochondrial function also abolished the effect of Didymin on cell inflammation (Fig. 6C, D) and apoptosis (Fig. 6E). In order to find out the target of Didymin, we conducted docking analysis to detected the interaction between Didymin and NRF1 protein. There are multiple groups of residues used to form interactions between receptor protein and ligand, such as the hydrogen bond formed by ALA73 of NRF1 and ligand. With these interaction forces, the binding energy of protein-ligand complex was − 6.5 kcal/mol, which is a good performance (Fig. S7). The results indicate that Didymin can regulate mitochondrial DNA transcription through NRF1, and then regulate mitochondrial function. These findings suggest that Didymin attenuates inflammation, improves GSIS function and inhibits apoptosis by enhancing mitochondrial function.

**Fig. 7:**
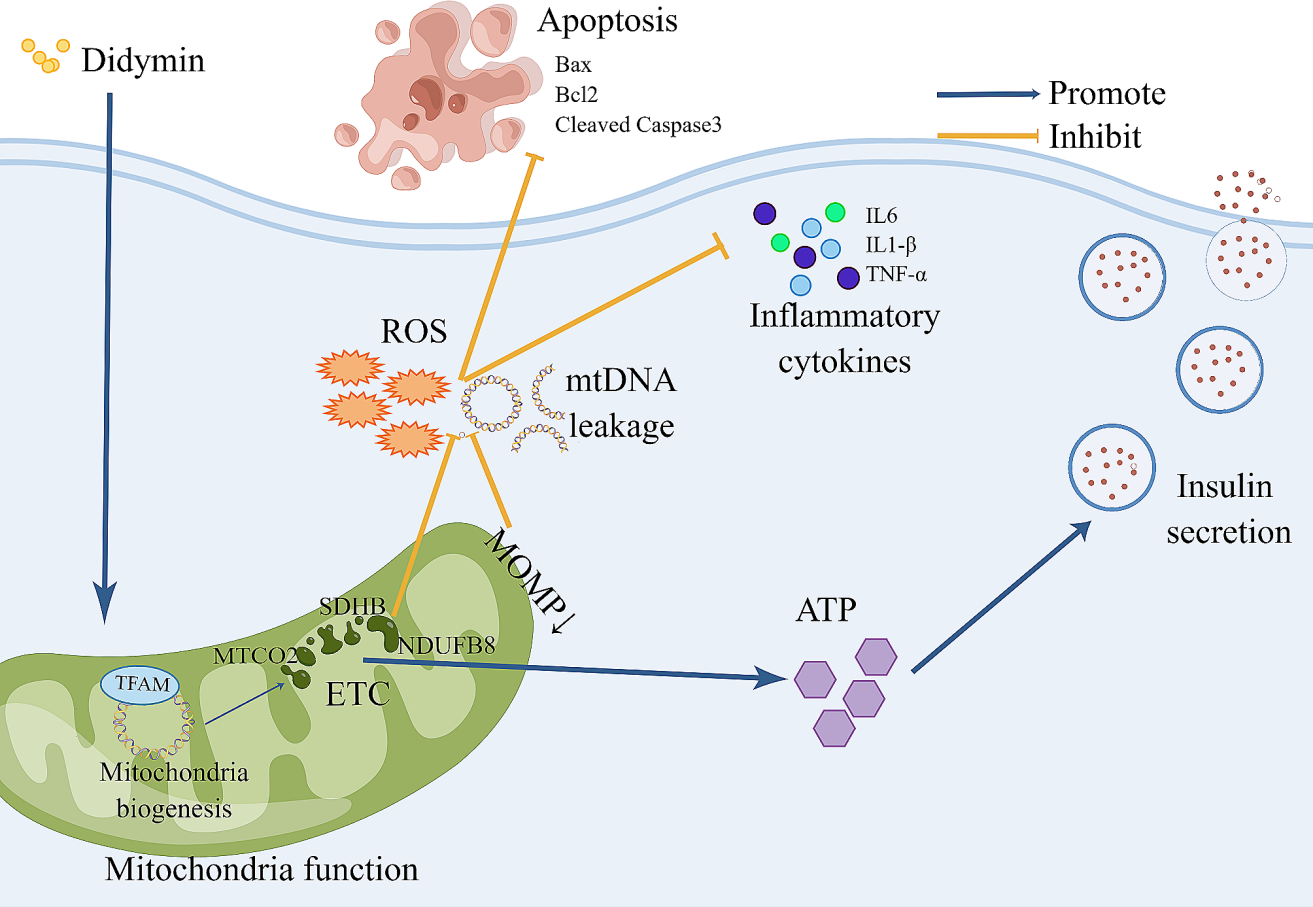
Schematic representation of Didymin alleviates IGT by enhancing mitochondria function

Overall, these evidences presented support the notion that Didymin can improve PBC function by enhancing mitochondrial function, inhibiting inflammation and attenuating apoptosis (Fig. 7).

## Discussion

In this study, we demonstrated that Didymin attenuates inflammation, improves PBC insulin secretion function and inhibits apoptosis by enhancing mitochondrial function both in PA-induced INS-1 cells and PBCs in HFD-induced IGT mice. These results suggest that Didymin could be a potential candidate for the treatment of HFD-induced IGT.

Initially, we observed that treatment with Didymin in IGT mice reduced postprandial glycemia and increased 30-minute postprandial insulin levels (Fig. 1), indicating an improvement in early insulin response. We observed the insulin content of islet beta cells by electron microscopy and pancreatic section staining, and the results showed that HFD mice have less insulin content in fasting state. In INS-1 cells, we evaluated insulin secretion function through GSIS, intracellular insulin content after GSIS, and transcription level of Insulin (Fig. 1). The results showed that the insulin synthesis of INS-1 cells did not change significantly after Didymin administration, but the insulin secretion of INS-1 cells increased after high glucose stimulation, and the intracellular insulin content decreased after high glucose stimulation. The results of in vitro and in vivo experiments showed that under high-fat stimulation, Didymin did not increase insulin synthesis of PBCs, but decreased insulin secretion and increased intracellular insulin content of PBCs under fasting (low glucose stimulation). Therefore, the first-phase insulin secretion can be more sufficient when stimulated by high glucose. The results demonstrated that Didymin improved PBC insulin secretion both in vitro and in vivo. We performed RNA sequencing to further explore the underlying mechanisms of Didymin in the treatment of IGT. The results indicated that mitochondria function, inflammation, and apoptosis are key pathways affected by Didymin (Fig. 2).

Next, we examined various aspects of mitochondrial function, including the number of mitochondria, expression levels of key mitochondrial proteins, oxidative respiration function, and intracellular ROS levels (Fig. 3). The results showed that the expression of NRF1 and TFAM, the key transcription factors regulating mitochondrial biosynthesis, were elevated, and the number of mitochondria increased, suggesting that the function of mitochondrial biosynthesis was improved. The levels of key proteins that constitute the mitochondrial oxidative respiratory chain were increased, the function of the mitochondrial oxidative respiratory chain was improved, and the production of ROS, a marker product of mitochondrial electron transport chain function damage, was reduced, indicating that Didymin can improve mitochondrial biogenesis and function. The results suggested that Didymin can enhance mitochondrial biogenesis, improve oxidative respiration function, and decrease intracellular ROS levels. Previous studies have also found that the increased mitochondrial biogenesis can improve mitochondria metabolism, respiratory function, and reduce ROS production [28], which are crucial for PBC function [29].

Subsequently, we investigated the effects of Didymin on inflammation and apoptosis. The inflammatory response of PBCs was evaluated by analyzing the protein and transcription levels of secreted cytokines IL6, IL1-β, and TNFα (Fig. 4). We also tested the activation of NF-κB pathway (Fig. S5), and the results show that Didymin inhibited the activation of NF-κB pathway stimulated by high fat and reduced the production of inflammatory factors. Apoptosis was evaluated through flow cytometric analysis, measurements of apoptosis related proteins, and the TUNEL/γ-H2AX assay (Fig. 5). TUNEL and /γ-H2AX staining showed that Didymin reduced the DNA breakage, Annexin-V/PI staining showed that Didymin reduced the damage of cell membrane integrity, and the decrease in the proportion of key proteins of apoptosis pathway suggested that Didymin inhibited PBC apoptosis. These results suggest that Didymin can inhibit inflammation and apoptosis, which are crucial for PBC function [30, 31].

In PBCs, long-chain FFAs, such as PA, are metabolized through β-oxidation in the peroxisomes as well as in the mitochondria [32]. Peroxisomal β-oxidation produces H_2_O_2_ [32], and in contrast with other tissues, peroxisomes in PBCs lack catalases, leading to the accumulation of ROS due to the inability to inactivate peroxisomal H_2_O_2_ [33]. Considering the close proximity of mitochondria to the source of ROS generation, mitochondrial components are highly susceptible to oxidative damage [34]. Mitochondrial DNA (mtDNA) encodes polypeptides of the electron transport chain (ETC) complexes and is more vulnerable than nuclear DNA to oxidative stress-related damage due to the lack of protective histones and limited repair mechanisms [34]. Therefore, mtDNA damage results in ETC dysfunction, leading to decreased in ATP synthesis. In addition to DNA damage, PBC mitochondria lose their fusion ability and become fragmented, causing mtDNA leakage [35]. MtDNA leakage, ATP deficiency, and excessive ROS accumulation contribute to cell inflammation, apoptosis, and impaired insulin secretion. Studies have proven that repairing mitochondrial damage is the key factor in improving cell function [36, 37]. Therefore, we further explored whether Didymin protects PBCs by regulating mitochondrial function.

To validate the role of mitochondria in regulating inflammation, apoptosis, and insulin secretion, we employed a mitochondria inhibitor (Rotenone). After the addition of inhibitor, we examined the glucose-stimulated insulin secretion function, the transcription and protein levels of inflammatory factors, and the levels of key proteins in the apoptotic pathway. The results showed that the addition of the inhibitor significantly inhibited the secretion of insulin, increased the level of inflammatory factors, and activated the apoptotic pathway. The results proved that inhibiting mitochondrial function prevents Didymin from improving inflammation, apoptosis, and insulin secretion (Fig. 6). Therefore, improving mitochondrial function is a key mechanism through which Didymin exerts its therapeutic effect.

In pancreatic beta cells, export of ATP to the cytosolic compartment promotes the closure of ATP-sensitive K^+^ channels (KATP-channel) on the plasma membrane, leading to cell depolarization [38]. This depolarization triggers Ca^2+^ influx, which is essential for insulin exocytosis [39]. Consequently, impaired mitochondrial function due to lipotoxicity-induced reduction in ATP production directly impairs GSIS [40]. In T2D patients, the first phase of insulin secretion is significantly abolished, suggesting a defect in priming and/or fusion of insulin-containing granules with the β-cell plasma membrane [41, 42]. These evidences support our study that GSIS can be improved by improving mitochondrial function when insulin gene transcription remains unaffected.

The generation of ROS by oxidative phosphorylation inhibitors can activate the inflammasome, while leaked mtDNA can stimulate various pattern recognition receptors (PRRs), including cGAS, TLR9 and NLRP3 inflammasomes, leading to the release of pro-inflammatory factors [43–47]. These studies corroborate our results that Didymin reduces the production of lipotoxicity-induced pro-inflammatory factors by improving mitochondrial function.

Patients with obesity and T2DM often exhibit a ~ 60% deficit in PBC mass, primary due to increased apoptosis [48]. Key proapoptotic members of the B cell lymphoma 2 (BCL-2) family, such as BAX and BAK, promote mitochondrial outer membrane permeabilization (MOMP) and initiate a signaling cascade that ultimately leads to cell death [25]. Moreover, FFAs cause dose-dependent damage in mtDNA, contributing to apoptosis [49, 50]. These observations confirmed our findings that Didymin ameliorates apoptosis induced by lipotoxicity in INS-1 cells, while this effect is counteracted by inhibiting mitochondrial function.

As mentioned above, HFD leads to mitochondrial dysfunction in PBCs, resulting in increased levels of inflammatory factors, impaired insulin secretion, and ultimately cell apoptosis. Other studies have also suggested that enhancing mitochondrial function can protect PBCs: increasing the expression of FUNDC1, a key regulatory protein of mitophagy, can reduce mitochondrial damage caused by lipotoxicity by increasing the level of mitophagy, thereby regulating insulin release and inhibiting cell apoptosis [51]. Overexpression of transcription factor STAT3 can improve mitochondrial biogenesis and mitochondrial function in PBCs of mice with high-fat diets, thereby improving insulin secretion [52]. Imeglimin, a tetrahydrotriazine-containing class of oral glucose-lowering agents enhances mitochondrial function in mice PBC by upregulating complex I protein expression. Furthermore, it inhibits endoplasmic reticulum stress and cell apoptosis and enhances insulin secretion [53]. Cholesterol sulfate, a sterol sulfate found in human plasma, can protect mitochondrial integrity, leading to a reduction in ROS generation and an increase in ATP production. This allows the insulin secretion mechanism in the pancreatic islets to function effectively and prevents cell apoptosis caused by lipotoxicity [54]. Therefore, improving mitochondrial function in the context of a high-fat diet is an effective target for improving PBC function.

Our GTT and ITT results showed that Didymin improved both glucose tolerance and insulin sensitivity in IGT mice (Fig. 1B, S1). These findings indicated that in addition to its effects on PBC, Didymin may also improve insulin sensitivity and glucose uptake in hepatocytes, myocytes and adipocytes. In our future studies, we aim to comprehensively explore the mechanisms by which Didymin improves glycemic control in various organs of IGT mice.

Furthermore, Our RNA sequencing results showed a significant change in the expression of exocytosis-related genes upon treatment with Didymin (Fig. S8). Previous studies have reported a notable decrease in the expression of exocytotic genes and proteins in islets from T2D donors [55]. This indicates that, in addition to its impact on mitochondrial pathway, Didymin may also exert a direct effect on insulin secretion. Further investigations are warranted to explore this potential mechanism. Our trial did not show significant side effects of Didymin. The behavior and appearance of the mice were normal during Didymin administration, and no mice died. Normal morphology of the remaining abdominal organs was observed during mouse dissection. There is also a lack of research on the side effects of Didymin in existing studies [13, 15, 16, 18, 56–60]. We will further investigate these questions in the following experiments. Our results show that didymin has a direct and significant improvement in mitochondrial function, so we speculate that it may play a great role in diseases where mitochondrial function is severely impaired. For example, mitochondrial encephalomyopathy [61], mitochondrial fatty acid oxidation deficiency [62], neurodegenerative diseases [63], myocardial ischemia, myocardial infarction [64], etc. In these diseases, Didymin may combine with existing therapies such as gene therapy [61], fat intake restriction [62], deep brain stimulation [63], antiplatelet drugs, and β-blockers [64] to improve disease symptoms. Hopefully, Didymin may serve as a safe and effective dietary-based therapy or food supplement following comprehensive clinical trials.

## Conclusion

Overall, our findings, for the first time, indicate that Didymin plays a vital role in alleviating IGT in mice. In vitro and in vivo experiments have demonstrated its ability to improve mitochondrial function in PBCs. Moreover, Didymin exhibits anti-inflammatory properties, improves GSIS and inhibits apoptosis by improving mitochondrial function. Collectively, our research concludes that Didymin is a dietary-based safe active component in functional foods for improving PBC function in HFD-induced IGT. The current study only focused on the impact of Didymin on PBCs’ function, but the research findings also suggest its potential to improve the function of other metabolic organs. We will comprehensively explore the effects of Didymin on various organs in future studies and thoroughly evaluate its side effects to investigate the possibility of its clinical application.

### Electronic supplementary material

Below is the link to the electronic supplementary material.

Supplementary Material 1

Supplementary Material 2

## Data Availability

All data are available from the corresponding author upon reasonable request.
