# Supplementary material for: Didymin protects pancreatic beta cells by enhancing mitochondrial function in high-fat diet-induced impaired glucose tolerance
Source: Diabetol Metab Syndr. 2024 Jan 3;16:7. doi: 10.1186/s13098-023-01244-1 (PMC10762818; doi:10.1186/s13098-023-01244-1)
Supplement: Supplementary file 2 — Supplementary Material 2 [file 13098_2023_1244_MOESM2_ESM.docx]

**Materials and methods:**

**1.** **The activity of NOX**

The activity of NOX was assayed using the commercially NOX activity assay kit (Solarbio, Beijing, China) following the manufacturer's instructions (Dou et al., 2017). The enzyme activity was expressed as units per milligram protein (U/g pro).

**2. Docking analysis**

The predicted structures of NRF1 were generated by Alphafold. The protonation state of all the compounds was set at pH = 7.4, and the compounds were expanded to 3D structures using Open Babel[1]. AutoDock Tools (ADT3) were applied to prepare and parametrize the receptor protein and ligands. The docking grid documents were generated by AutoGrid of sitemap, and AutoDock Vina (1.2.0) was used for docking simulation[2, 3]. The optimal pose was selected to analysis interaction. Finally, the protein-ligand interaction figure was generated by PyMOL. The NRF1 protein is represented as a slate cartoon model, ligand is shown as a cyan stick, and their binding sites are shown as magentas stick structures. Nonpolar hydrogen atoms are omitted. The hydrogen bond, ionic interactions, and hydrophobic interactions are depicted as yellow, magentas and green dashed lines, respectively.

**Supplement figures:**

**
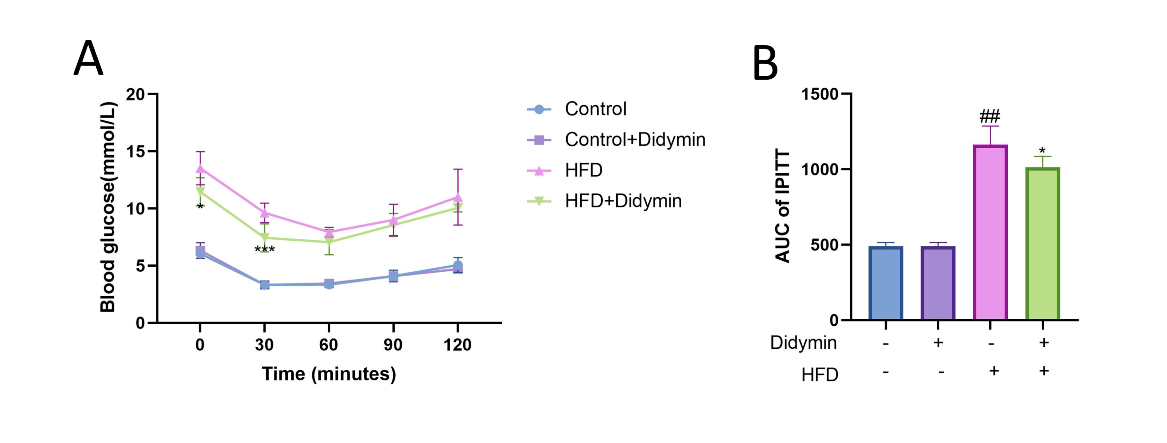
**

**Figure S1** Didymin improves insulin resistance in IGT mice. (A) Serum glucose level in IPITT. (B) AUC of IPITT.


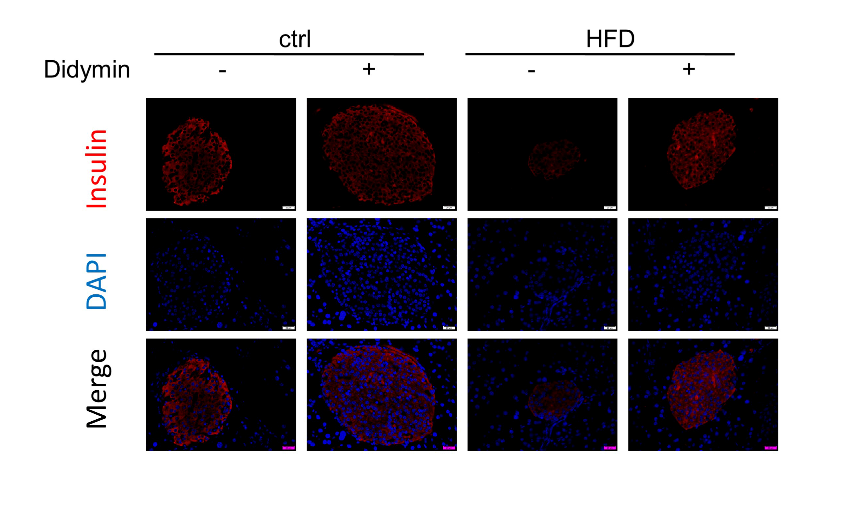


**Figure S2** Insulin staining of pancreatic sections.


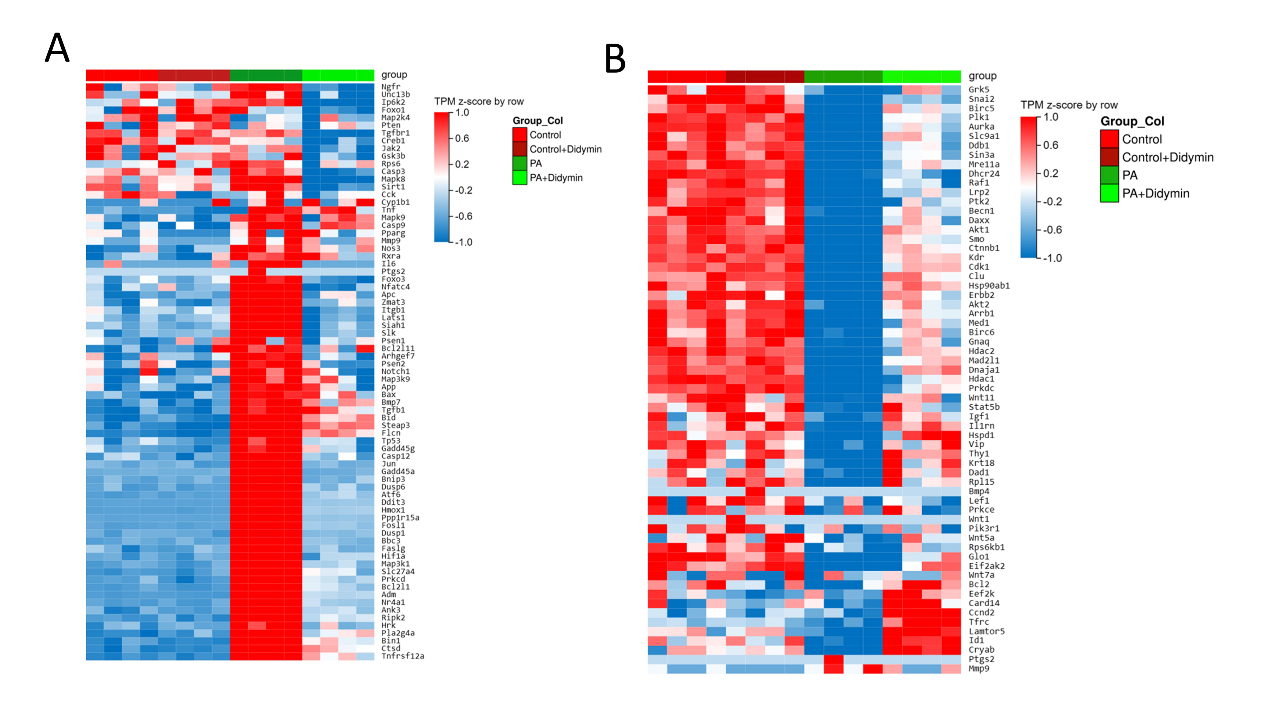


**Figure S3** Heatmap of genes in regulators of apoptosis affected by Didymin. (A) Positive regulators of apoptosis (B) Negative regulators of apoptosis.


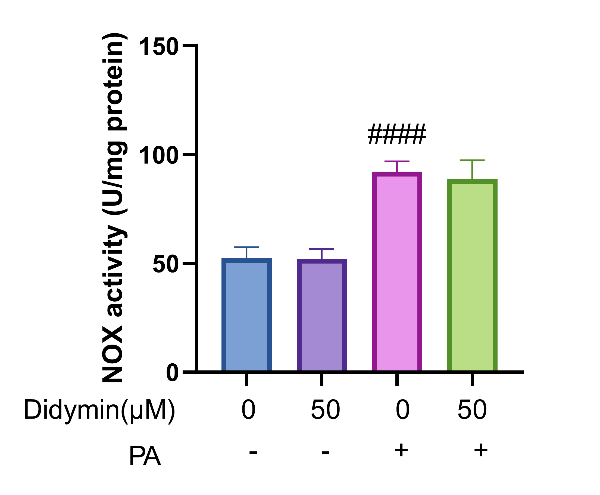


**Figure S4** NOX activity in INS-1 cells.


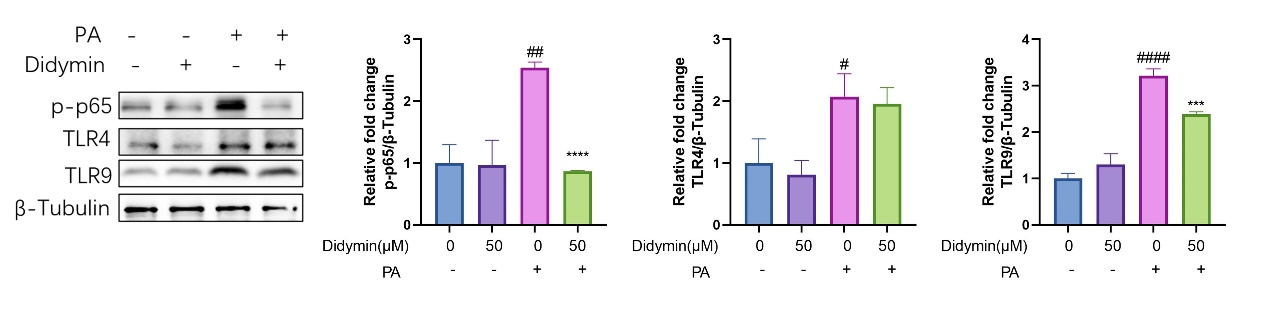


**Figure S5** Western blot analysis of the p-p65, TLR4 and TLR9 proteins in INS-1 cells.


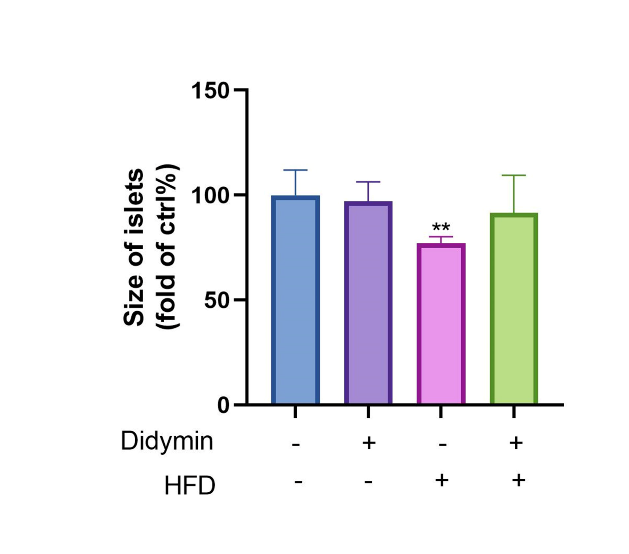


**Figure S6** Size of islets.


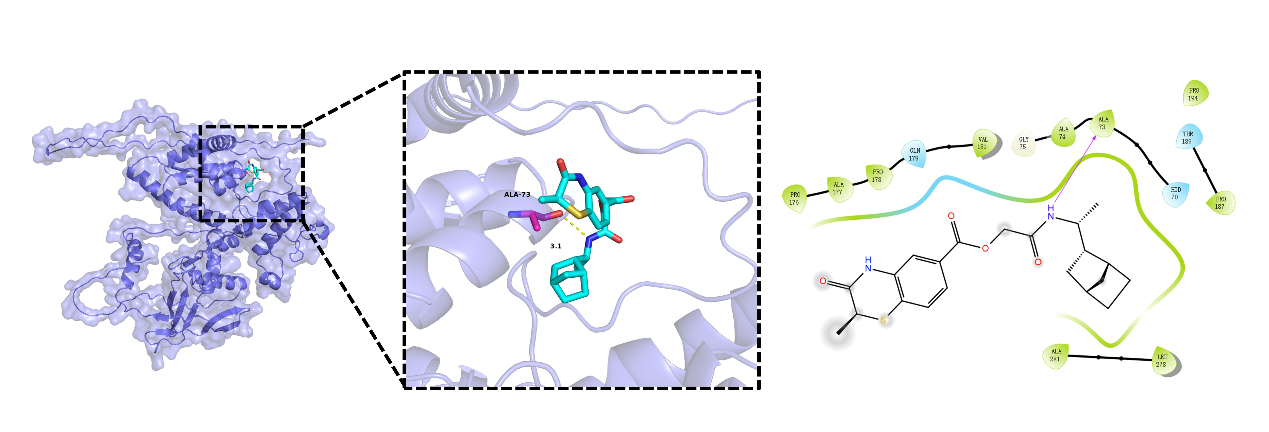


**Figure S7** Docking analysis of Didymin and NRF1 protein.

**
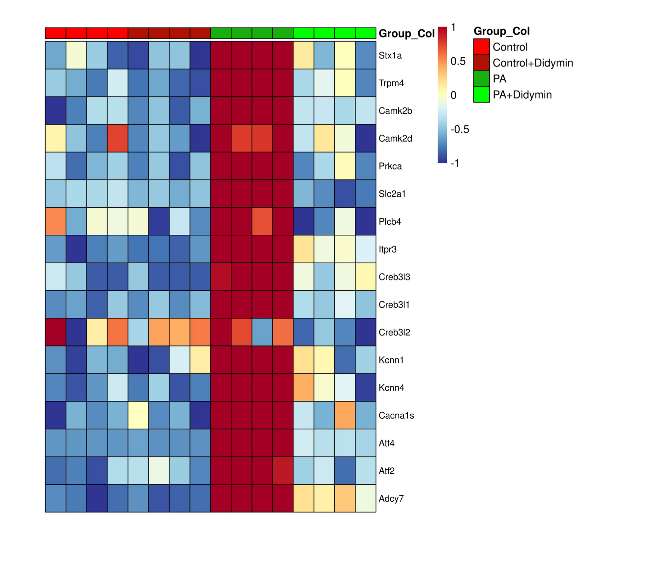
**

**Figure S8** Heatmap of genes in exocytosis pathway affected by Didymin.


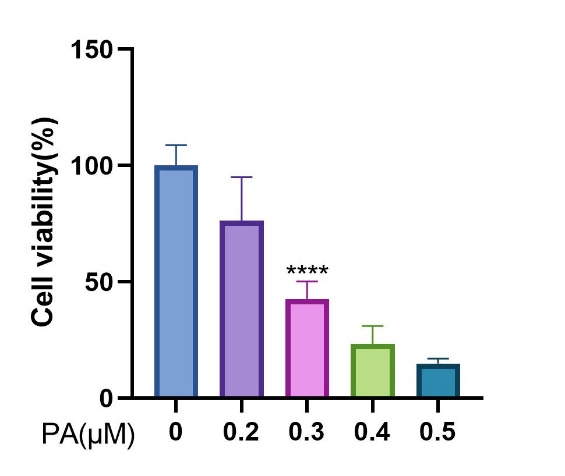


**Figure S9** Cell viability of INS-1 cells treated with different concentrations of PA. Data are expressed as mean ± SD (n = 4). ****P < 0.0001 Control vs. PA.

1. O'Boyle, N.M., M. Banck, C.A. James, C. Morley, T. Vandermeersch, and G.R. Hutchison, *Open Babel: An open chemical toolbox.* J Cheminform, 2011. **3**: p. 33.

2. Eberhardt, J., D. Santos-Martins, A.F. Tillack, and S. Forli, *AutoDock Vina 1.2.0: New Docking Methods, Expanded Force Field, and Python Bindings.* J Chem Inf Model, 2021. **61**(8): p. 3891-3898.

3. Trott, O. and A.J. Olson, *AutoDock Vina: improving the speed and accuracy of docking with a new scoring function, efficient optimization, and multithreading.* J Comput Chem, 2010. **31**(2): p. 455-61.
